# Hyaluronic Acid-Based Nanoplatforms for Photoacoustic Monitoring of Phototherapy

**DOI:** 10.3390/biom16101509

**Published:** 2026-10-17

**Authors:** Yongjae Jeong, Dongyoung Jo, Wonseok Choi, Ki Su Kim, Jeesu Kim

**Affiliations:** 1 Department of Cogno-Mechatronics Engineering, School of Transdisciplinary Engineering, Pusan National University, Busan 46241, Republic of Korea; zxc2726655@pusan.ac.kr (Y.J.); jdy9321@pusan.ac.kr (D.J.); 2 Department of Biomedical Engineering and Medical Sciences, College of Medicine, The Catholic University of Korea, Seoul 06591, Republic of Korea; wonseok.choi@catholic.ac.kr; 3 Department of Bioengineering, College of Engineering, Hanyang University, Seoul 04763, Republic of Korea

**Keywords:** photoacoustic imaging, hyaluronic acid, photothermal therapy, photodynamic therapy

## Abstract

Photoacoustic imaging is a noninvasive imaging modality with considerable potential for theranostic applications, providing high-resolution visualization of molecular and physiological responses to treatment. Among the diverse materials investigated for constructing theranostic nanoplatforms, hyaluronic acid has attracted substantial interest because of its biocompatibility, hydrophilicity, chemical modifiability, and affinity for CD44 receptors, which are frequently overexpressed in various cancer cells. Moreover, appropriately engineered hyaluronic acid-based formulations offer opportunities for localized and transdermal delivery, potentially reducing the need for systemic administration and expanding the applicability of noninvasive phototherapy, particularly for superficial lesions. In this review, we summarize recent advances in hyaluronic acid-based nanoplatforms for photoacoustic imaging-monitored phototherapy, categorized into photothermal therapy, photodynamic therapy, and their combination. We further discuss their material design, targeting and delivery mechanisms, photoacoustic monitoring capabilities, therapeutic performance, and key challenges for clinical translation.

## 1. Introduction

Theranostics is an approach that integrates therapy and diagnosis for effective disease management [1,2,3,4]. Biomedical imaging plays a central role in theranostic applications by enabling the visualization of disease targets, evaluation of therapeutic agent distribution, and monitoring treatment responses. Conventional biomedical imaging modalities, such as magnetic resonance imaging (MRI) [5,6], positron emission tomography (PET) [7,8], and X-ray computed tomography (CT) [9,10], provide complementary anatomical, functional, and molecular information. However, their relatively high cost, limited portability, and the use of ionizing radiation (PET and CT) may restrict their utility in longitudinal studies. In contrast, ultrasound (US) imaging [11,12,13] and optical imaging techniques [14,15] are attractive for repeated examination due to their relatively low cost, ease of operation, and being free from ionizing radiation. US imaging techniques provide real-time, high-resolution monitoring of therapy-induced tissue alterations, including tissue morphology, blood flow power and velocity, and tissue stiffness, but have limited capability for molecular differentiation of tissue composition. Optical imaging modalities, such as fluorescence imaging (FLI) [16,17] and two-photon microscopy (TPM) [18,19], have been extensively used for monitoring therapeutic responses following phototherapy, such as photothermal therapy (PTT) [20,21,22] and photodynamic therapy (PDT) [23,24,25]. Nevertheless, the imaging depth of these techniques is generally limited by strong optical scattering in biological tissues.

In this sense, photoacoustic imaging (PAI) is a promising imaging modality that combines the advantages of optical absorption contrast and US spatial resolution, enabling deeper tissue imaging compared to pure optical imaging techniques [26,27,28,29]. PAI is based on the photoacoustic (PA) effect, in which the absorbed pulsed light energy induces a transient temperature rise and thermoelastic expansion, thereby generating acoustic waves [30,31,32]. The initial pressure (P) of the generated PA waves is expressed as follows:

(1) $$P\propto \Gamma \left(T\right)\;\times \;\sigma \;\times \;\mu_{a}\;\times \;F,$$

where Γ is the Grüneisen parameter depending on the local temperature (T), σ is the thermal conversion efficiency, $\mu_{a}$ is the optical absorption coefficient, which depends on the chemical composition and light wavelength, and F is the local optical fluence. The generated acoustic waves are detected using US transducers and reconstructed to visualize the spatial distribution of optical absorbers.

PAI can obtain label-free imaging of endogenous chromophores by visualizing optical absorption from multispectral light illumination [33,34,35,36]. For example, differences in the optical absorption spectra of oxy-hemoglobin (HbO) and deoxy-hemoglobin (HbR) can be used to estimate oxygen saturation (sO_2_) level, providing functional information on tissue oxygenation [37,38,39,40]. Beyond label-free imaging, exogenous agents have also been widely used for PAI to enhance optical contrast in deeper biological tissue [41,42,43,44,45]. Using such agents, PAI has been widely applied to monitor in vivo drug delivery and to assess therapeutic responses during phototherapy [46,47,48,49]. PAI can also visualize the delivery and accumulation of photothermal agents or photosensitizers because the absorbed optical energy can be dissipated through heat generation or photochemical reactions [50,51,52].

The therapeutic efficacy of both PTT and PDT is strongly influenced by the selective delivery of therapeutic agents in target tissues. Accordingly, various carrier systems, including inorganic nanoparticles [53,54], carbon-based materials [55], and polymer-based carriers [56,57], have been extensively researched. In addition, biomolecule-based carriers such as liposomes, exosomes, antibodies, and peptides have been widely investigated because of their biocompatibility and specific biological recognition [58,59,60,61]. Among them, hyaluronic acid (HA)-based nanoplatforms have attracted considerable attention because HA can serve as a biocompatible and chemically modifiable polymeric component for tumor targeting, surface functionalization, and therapeutic agent delivery. HA is a major component of the human extracellular matrix (ECM) and exhibits specific binding affinity toward CD44 receptors, which are frequently overexpressed in various cancer cells [62,63,64]. Furthermore, HA can be incorporated into various hybrid nanoplatforms containing photothermal agents, photosensitizers, inorganic nanoparticles, metal–organic frameworks, or carbon-based materials. This structural versatility makes HA highly suitable for PA monitoring of phototherapeutic responses.

Several reviews have discussed HA-based nanoparticles for cancer therapy [65,66,67] or PA theranostic agents for phototherapy [68,69,70] from broader material and imaging perspectives. However, the specific integration of HA-mediated delivery with PA monitoring of phototherapy has received comparatively limited attention. In particular, the practical use of PAI for assessing the accumulation of HA-based phototherapeutic agents, selecting irradiation timing, and comparing systemic and localized delivery strategies has not been emphasized. This review therefore focuses on HA-based nanoplatforms for PA-monitored PTT, PDT, and combined PTT/PDT. First, we introduce the fundamental principles of phototherapy and HA-based nanoplatforms. Then, we discuss representative HA-based platforms according to their therapeutic mechanisms and PAI strategies. Finally, we discuss current limitations and future perspectives for the development of HA-based PA therapeutic platforms.

## 2. Fundamentals of Phototherapy and Hyaluronic Acid-Based Nanoplatform

**Figure 1:**
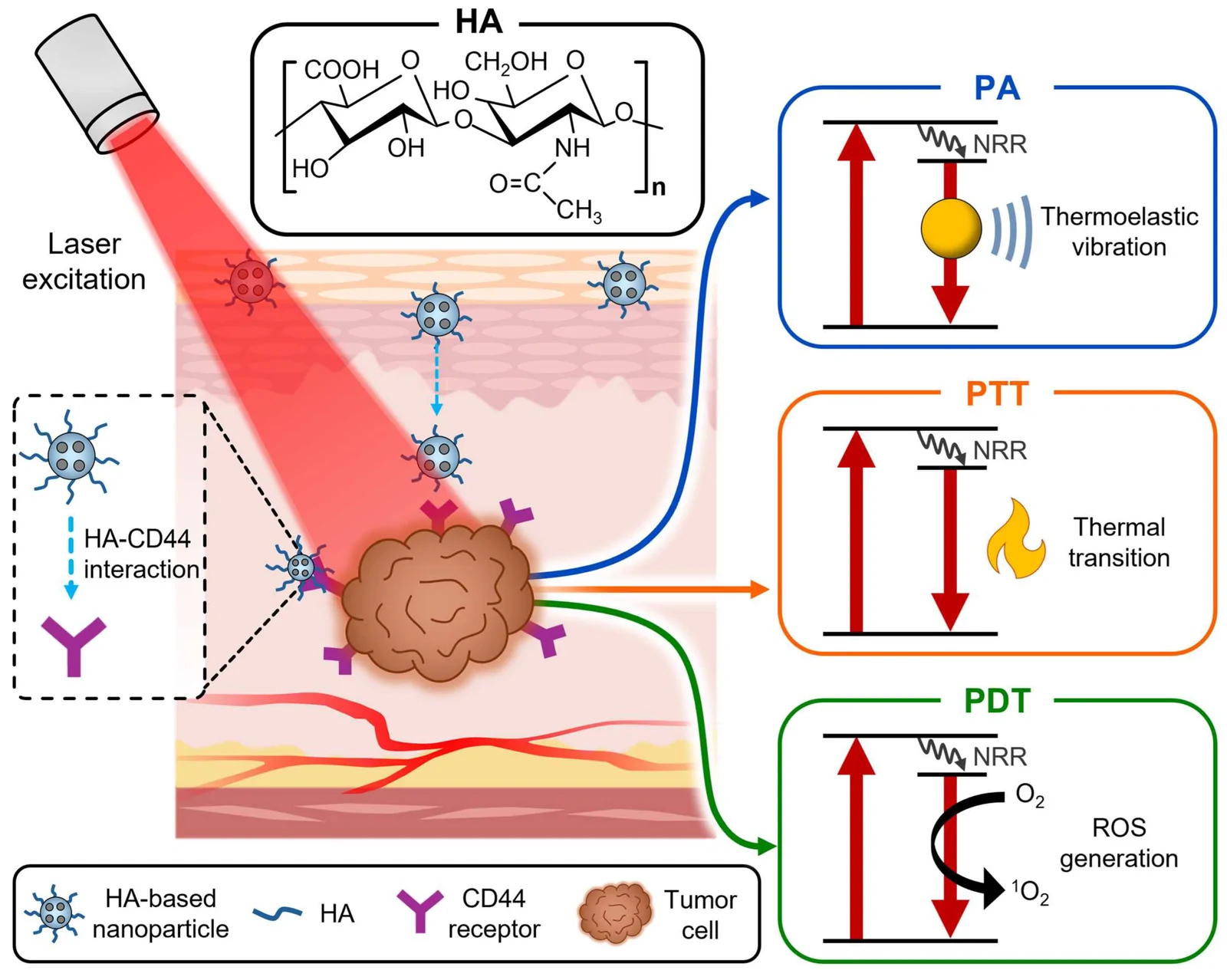
Schematic illustration of the principles of photoacoustic imaging, photothermal therapy, and photodynamic therapy mediated by hyaluronic acid-based nanoplatforms. PA, photoacoustic; PTT, photothermal therapy; PDT, photodynamic therapy; HA, hyaluronic acid; ROS, reactive oxygen species; NRR, nonradiative relaxation; O_2_, oxygen; ^1^O_2_, singlet oxygen.

PTT and PDT both begin with the absorption of optical energy by an exogenous agent but differ in how the absorbed energy is subsequently dissipated (Figure 1). After excitation to the excited singlet state, the agent releases the absorbed energy through competing pathways [71,72]. Internal conversion nonradiatively converts the energy into heat, which underlies both PTT and PA signal generation, whereas fluorescence emits it as light. Alternatively, intersystem crossing to the triplet state enables energy or electron transfer to molecular oxygen, generating reactive oxygen species (ROS) for PDT. The relative contributions of these pathways are primarily determined by the quantum yields of internal conversion, fluorescence, and intersystem crossing, which are intrinsic to the agent, and can be further modulated by external conditions such as aggregation and oxygen availability.

In PTT, therapeutic efficacy is primarily determined by the amount of photothermal agent accumulated at the target, its optical absorption and photothermal conversion efficiency, and the delivered light dose. Localized heating can induce protein denaturation, membrane disruption, vascular injury, and irreversible thermal damage to diseased tissue. Accordingly, targeted delivery is important for achieving sufficient heating at the lesion while minimizing thermal damage to adjacent normal tissue.

In PDT, the triplet-state photosensitizer generates ROS through Type I electron- or hydrogen-transfer reactions or Type II energy transfer to molecular oxygen. These reactive species induce oxidative damage to cellular components and tumor-associated vasculature. Therefore, PDT efficacy depends not only on photosensitizer accumulation and light dose but also on local oxygen availability, which can become limiting in hypoxic tumors or during irradiation.

HA is a naturally occurring linear glycosaminoglycan composed of repeating disaccharide units of D-glucuronic acid and N-acetyl-D-glucosamine [73]. Its carboxyl and hydroxyl groups provide reactive sites for chemical modification and conjugation, allowing HA to be incorporated into micelles, nanogels, hydrogels, microneedles, and hybrid nanoparticles [74,75,76,77]. Hydrophobic photothermal agents or photosensitizers can be conjugated to HA, encapsulated within HA-derived assemblies, or combined with HA-coated nanostructures. In HA-coated nanostructures, the hydrophilic HA chains form a hydrated layer on the particle surface, which reduces particle aggregation and keeps the particles well dispersed in aqueous solution [78]. In HA-derived assemblies, poorly water-soluble phototherapeutic agents are held within the hydrophobic core, which can reduce their premature release during delivery [79,80]. As a result, HA-based nanoplatforms can improve the aqueous dispersibility and colloidal stability of phototherapeutic agents.

The biological behavior of HA-based nanoplatforms is influenced by factors such as HA molecular weight, surface density, particle size, and receptor accessibility. Interaction between HA and CD44 can promote cellular binding in tumors with elevated CD44 expression, which is often observed in various cancer cells. Following cellular uptake, degradation of HA by hyaluronidases can destabilize the carrier and facilitate the release or activation of incorporated agents. Thus, HA functions not only as a targeting component but also as a delivery matrix that supports the effective application of PTT and PDT.

When optically absorbing phototherapeutic agents are incorporated into HA-based nanoplatforms, PAI can be performed at the same optical wavelength used for phototherapy despite the differences in the laser irradiation patterns, enabling direct visualization and quantitative estimation of the therapeutic efficacy of nanoplatforms. At the same time, PAI can provide spatially resolved images of surrounding tissue and vasculature, potentially useful for evaluating the responses and progression status of the treated tissue [69,81]. The specific roles of PA monitoring in HA-based PTT, PDT, and combined phototherapy are discussed in the following sections.

## 3. Photoacoustic Monitoring of Hyaluronic Acid-Based Nanoplatforms for Photothermal Therapy

Photothermal agents exhibit strong optical absorption, which not only enables efficient heat generation but also produces PA signals. This dual functionality allows PAI to monitor the delivery and accumulation of HA-based photothermal nanoplatforms before therapeutic irradiation. An increase in PA signal intensity at the target site can provide an indication of successful agent delivery and may assist in determining an appropriate time point for therapeutic irradiation. Beyond pre-treatment localization, PAI can also monitor treatment-associated physiological responses following PTT, including changes in vascular perfusion, vessel integrity, hemoglobin concentration, and blood oxygen saturation. Therefore, PAI provides a useful tool for monitoring both nanoplatform delivery and therapeutic effects in HA-based PTT (Table 1).

**Figure 2:**
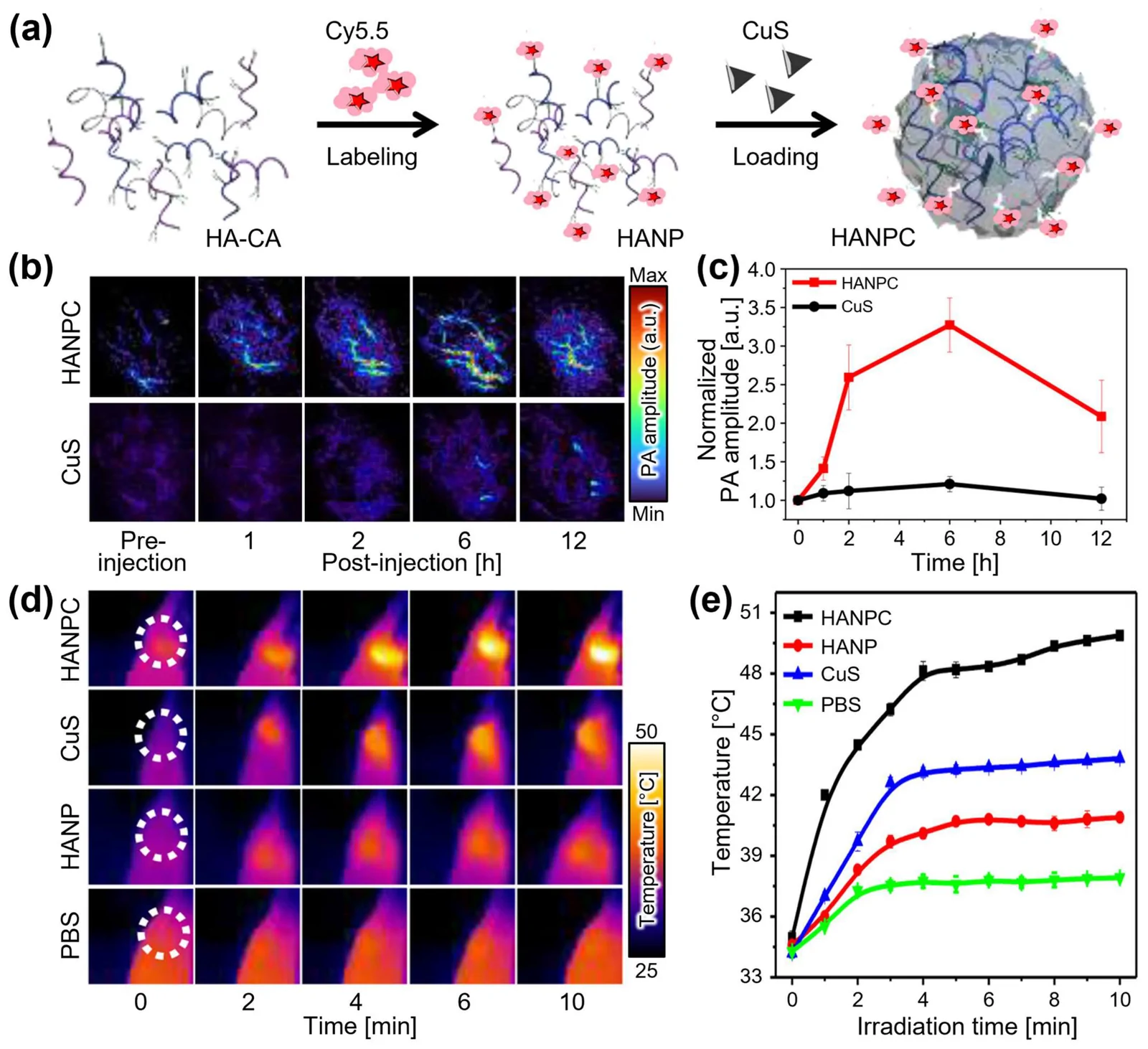
HA-based CuS nanocomposite for PA-monitored PTT. (**a**) Schematic illustration of the preparation of HANPC by loading CuS into HA nanoparticles. (**b**) PA images of tumors in mice before and after injection of HANPC and free CuS. (**c**) Normalized PA amplitude in the tumor region, showing enhanced accumulation of HANPC and a maximum PA signal at 6 h post-injection. (**d**) Infrared thermal images during laser irradiation. (**e**) Temperature profiles at the tumor region (white dashed circle in (**d**)), showing a greater temperature increase in the HANPC-treated group than in the control groups. HA, hyaluronic acid; PA, photoacoustic; PTT, photothermal therapy; HA-CA, HA-cholanic acid conjugate; HANP, Cy5.5-labeled HA nanoparticle; CuS, copper sulfide; HANPC, HANP loaded with CuS; PBS, phosphate-buffered saline. The images are reproduced with permission from Ref. [82].

Copper sulfide (CuS) has been widely investigated as a material for PTT and PAI owing to its low cost, low cytotoxicity, and excellent optical and electrical properties [88,89,90]. However, free CuS has limited applicability in cancer diagnosis and therapy because of its poor tumor-targeting ability. To address this limitation, Zhang et al. developed a HA-based nanocomposite, denoted as HANPC, by first conjugating the fluorescence dye Cy5.5 to HA nanoparticles and subsequently loading CuS (Figure 2a) [82]. In this system, HA nanoparticles served as tumor-targeting carriers for the selective delivery of CuS to cancer cells overexpressing CD44 receptors, while CuS acted as both a PA contrast agent and a photothermal agent owing to its strong NIR absorption. The HA-based nanocarrier also improved the aqueous stability of CuS and promoted tumor accumulation through HA-mediated active targeting as well as passive targeting via the enhanced permeability and retention effect. After intravenous injection of HANPC into tumor-bearing mice, time-dependent PAI showed gradual accumulation of HANPC at the tumor site, whereas free CuS produced only weak PA signals (Figure 2b). Quantitative PA analysis further showed that the tumor PA signal reached its maximum at 6 h post-injection, suggesting an appropriate time point for therapeutic irradiation (Figure 2c). Accordingly, 808 nm NIR laser irradiation was performed at 6 h post-injection with a power density of 1.5 W/cm^2^ for 10 min. Infrared thermal imaging confirmed efficient heat generation in the HANPC-treated group (Figure 2d), with the temperature reaching approximately 50 °C after 4 min and exceeding that observed in the CuS, Cy5.5-labeled HA nanoparticle, and phosphate-buffered saline (PBS) groups (Figure 2e). Although these approaches require invasive injection, and the injected nanoparticles must pass through the systemic circulation before reaching the tumor, results demonstrate that HA-based delivery can improve the tumor localization of photothermal agents.

**Figure 3:**
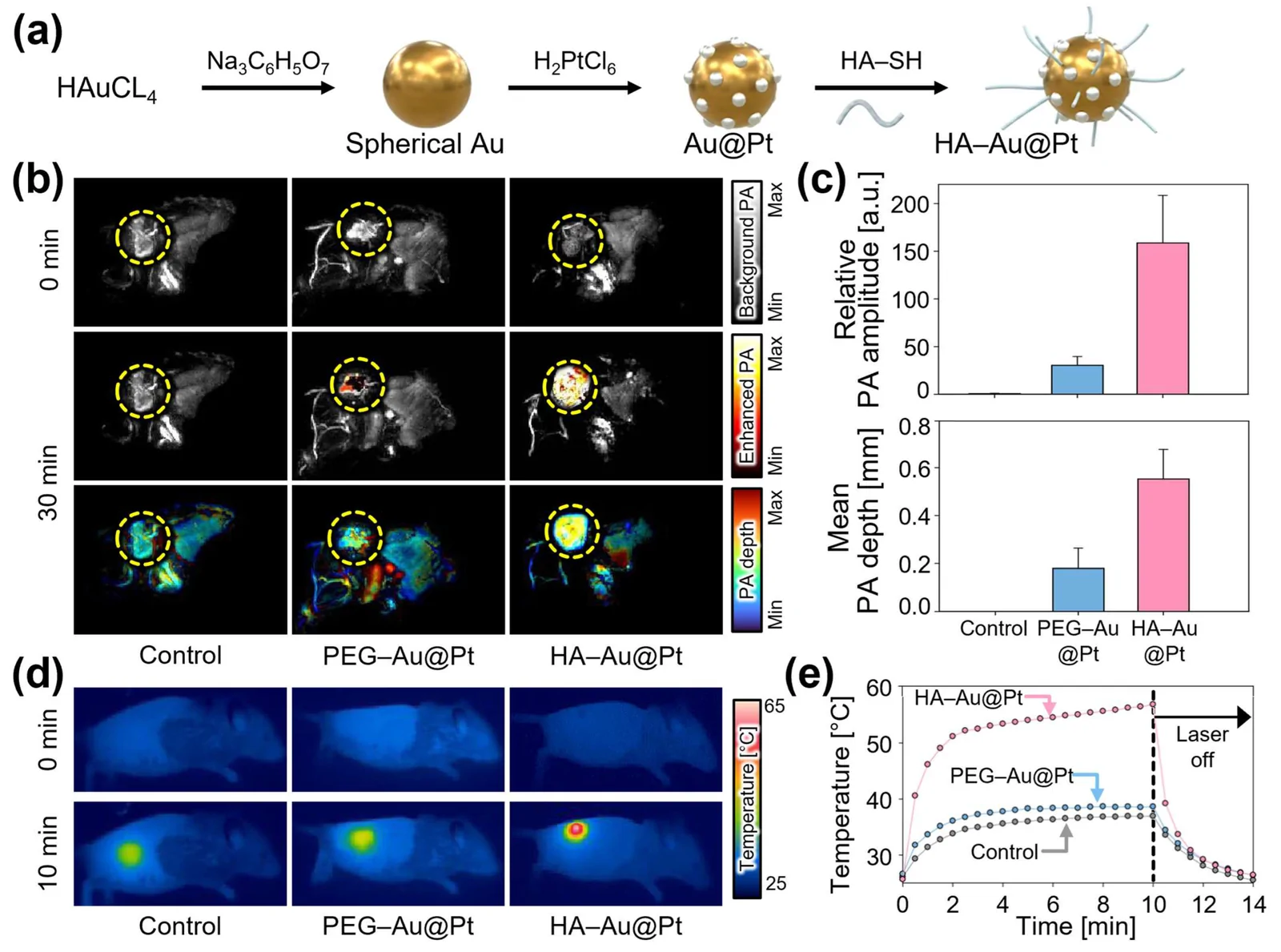
HA-based nanoparticles for transdermal PA-monitored PTT. (**a**) Schematic illustration of the preparation of HA-Au@Pt nanoparticles by Pt deposition onto spherical Au nanoparticles followed by surface modification with thiolated HA. (**b**) PA images of B16F10 tumors after transdermal delivery of HA-based nanoparticles. The tumor regions are indicated by yellow dashed circles. (**c**) Quantitative comparison of relative PA amplitude and PA penetration depths. (**d**) Infrared thermal images and (**e**) corresponding temperature profiles during 808 nm laser irradiation. HA, hyaluronic acid; PA, photoacoustic; PTT, photothermal therapy; Au, gold; HA-Au@Pt, HA-modified gold-platinum; Au@Pt, gold-platinum; PEG-Au@Pt, polyethylene glycol-modified gold-platinum. The images are reproduced with permission from Ref. [83].

While most PTT platforms have been designed to deliver photothermal agents to tumor sites through intravenous injection, transdermal delivery through the skin surface may provide an attractive alternative for externally accessible lesions such as skin tumors [91,92]. Han et al. developed a noninvasive theranostic platform using hyaluronate-modified gold-platinum (HA-Au@Pt) nanoparticles, in which HA was used to facilitate transdermal delivery and tumor targeting for PTT [83]. The nanoparticles were fabricated by growing platinum nanodots on the surface of spherical gold nanoparticles to form gold-platinum (Au@Pt) structures, followed by conjugation with thiolated HA via a gold–thiol reaction (Figure 3a). In this system, platinum nanodots enhanced NIR absorption and photothermal conversion through the surface plasmon resonance coupling effect, whereas HA contributed to skin penetration and tumor localization. After topical application of HA-Au@Pt nanoparticles to tumor-bearing mice for 30 min, PAI at 808 nm showed a marked increase in signal within the tumor region (Figure 3b). HA-Au@Pt nanoparticles produced approximately 5.23-fold higher PA amplitude than polyethylene glycol-modified gold-platinum (PEG-Au@Pt) nanoparticles and showed a mean penetration depth of 0.55 ± 0.12 mm, approximately 3.08-fold deeper than that of PEG-Au@Pt nanoparticles (Figure 3c). These results indicate that HA modification effectively enhanced transdermal penetration and tumor accumulation. Subsequent 808 nm NIR laser irradiation at 1.5 W/cm^2^ for 10 min produced a rapid temperature increase at the tumor site (Figure 3d), reaching approximately 56 °C in the HA-Au@Pt treated group, whereas the control and PEG-Au@Pt treated group showed substantially lower heating (Figure 3e). While the long-term safety needs to be evaluated since the Au@Pt is not biodegradable and may remain in the body for a long time, these results demonstrate the extension of HA-based PA-monitored PTT from systemic administration to localized thermal delivery.

**Figure 4:**
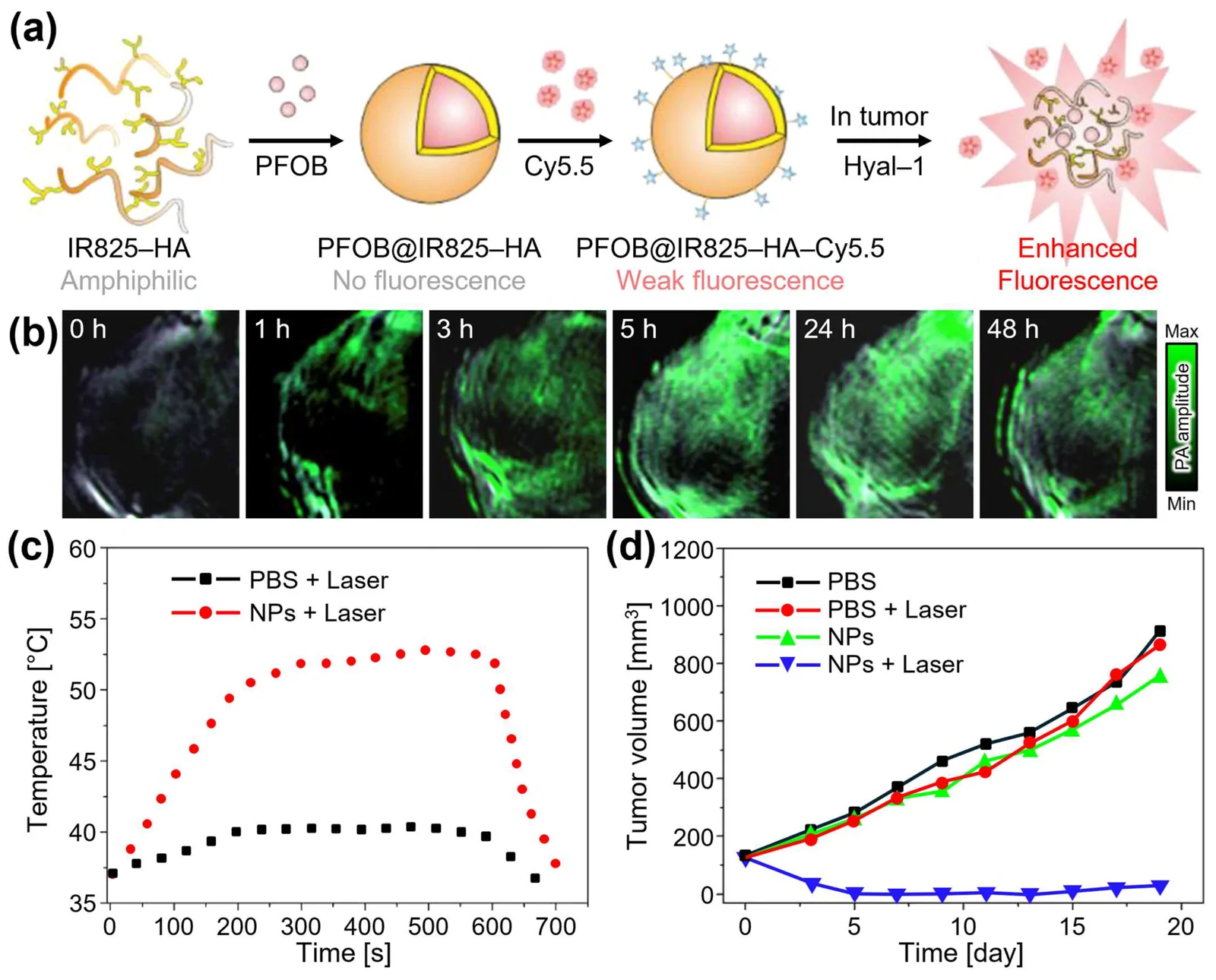
Hyaluronidase-responsive HA-based theranostic nanoplatform for multimodal imaging and PTT. (**a**) Schematic illustration of nanoparticle preparation and Hyal-1-mediated fluorescence activation. (**b**) Time-dependent PA images after nanoparticle injection, demonstrating increased PA contrast in the tumor region. (**c**) Temperature profiles during 808 nm laser irradiation. (**d**) Tumor growth curves following different treatments. PTT, photothermal therapy; PA, photoacoustic; HA, hyaluronic acid; PFOB, perfluorooctyl bromide; Hyal-1, hyaluronidase-1; PBS, phosphate-buffered saline; NP, nanoparticle. The images are reproduced with permission from Ref. [84].

Beyond single-modal monitoring, HA-based PTT platforms have also been developed as multimodal theranostic systems to improve tumor localization and treatment planning [93,94]. In this context, Liang et al. developed activatable nanoparticles that integrate fluorescence imaging (FLI), CT, PAI, and PTT within a single platform [84]. The nanoparticle was fabricated by encapsulating perfluorooctyl bromide (PFOB) inside IR825-conjugated HA nanoparticles and further conjugating Cy5.5 to the nanoparticle surface (Figure 4a). In the proposed nanoparticle, PFOB served as a CT contrast agent, IR825 functioned as both a PA contrast agent and a photothermal agent, and Cy5.5 fluorescence was designed to increase following HA degradation by tumor-associated hyaluronidase-1. After intravenous injection of the nanoparticles into tumor-bearing mice, FLI showed progressive tumor accumulation, with strong contrast observed at 24 h post-injection. CT imaging also showed enhanced contrast in the tumor region after injection, confirming nanoparticle accumulation within the tumor. In PAI, the PA signal gradually increased after injection and reached its maximum at 24 h post-injection (Figure 4b). Based on these complementary imaging results, 24 h post-injection was selected for 808 nm laser irradiation. The nanoparticle-treated group showed rapid tumor heating to approximately 50 °C (Figure 4c) and marked suppression of tumor growth compared to control groups (Figure 4d). While substantial nanoparticle accumulation was also observed in the liver, highlighting a limitation of systemic HA-based delivery, multimodal imaging enabled simultaneous assessment of off-target biodistribution and tumor accumulation.

In addition, HA has been combined with various other photothermal agents. Phan et al. coated iron–platinum nanoparticles with HA and used PAI to visualize the particles within the tumor [85]. In some platforms, the HA-based photothermal agent also carried a drug or a gene. Zhao et al. loaded docetaxel into a phase change material core surrounded by an HA-modified polypyrrole shell [86]. The polypyrrole shell generated both heat and PA signals, and the heat melted the core to release the drug. Liu et al. used HA-cloaked gold nanorods to deliver the p53 gene [87]. PAI showed that the HA cloak increased the accumulation of the nanorods in the tumor, and laser irradiation at the time of peak PA signal effectively suppressed tumor growth. These studies show that HA can be combined with a wide range of photothermal materials, from inorganic nanoparticles to conducting polymers, and with additional drug or gene payloads.

## 4. Photoacoustic Monitoring of Hyaluronic Acid-Based Nanoplatforms for Photodynamic Therapy

Photosensitizers used in PDT can provide PA contrast or be combined with additional PA-active components, enabling PAI to assess their delivery and accumulation before therapeutic irradiation. Because PDT efficacy depends on sufficient photosensitizer localization at the target site, time-dependent PAI can provide information on nanoplatform accumulation and retention and help identify an appropriate time point for light irradiation (Table 2).

**Figure 5:**
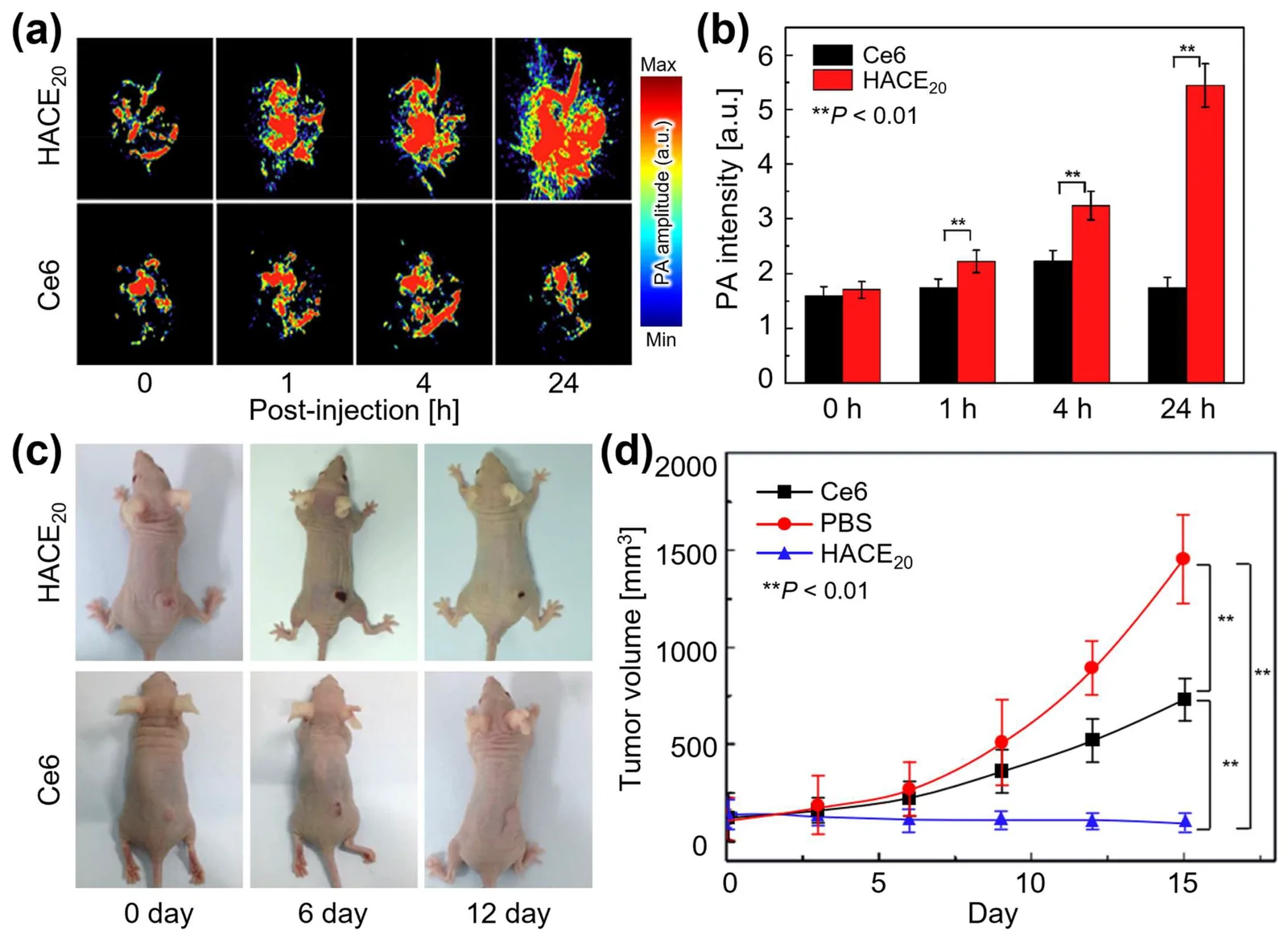
HA–Ce6 conjugate nanoparticles for PA-monitored PDT. (**a**) Time-dependent in vivo PA images of A549 tumors after intravenous injection of HACE_20_ nanoparticles or free Ce6. (**b**) Quantitative PA intensity in the tumor region at different post-injection time points, showing enhanced tumor accumulation of HACE_20_ nanoparticles. (**c**) Representative photographs of A549 tumor-bearing mice during treatment with HACE_20_ nanoparticles or free Ce6. (**d**) Tumor growth curves of mice treated with HACE_20_ nanoparticles, free Ce6, or PBS, demonstrating enhanced tumor suppression by the HACE_20_ nanoplatform. HACE_20_, HA–ADH–Ce6 conjugate nanoparticles prepared using 20 kDa HA; HA, hyaluronic acid; ADH, adipic dihydrazide; Ce6, chlorin e6; PA, photoacoustic; PDT, photodynamic therapy; PBS, phosphate-buffered saline. The images are reproduced with permission from Ref. [95].

A straightforward strategy for integrating HA with PDT is to directly conjugate a photosensitizer to the HA backbone. Li et al. developed self-assembled nanoparticles named HACE by coupling chlorin e6 (Ce6) to HA through an adipic dihydrazide (ADH) linker [95]. The conjugation of hydrophobic Ce6 to hydrophilic HA produced amphiphilic conjugates that spontaneously formed nanoparticles in aqueous solution. Among formulations prepared with different HA molecular weights, nanoparticles based on 20 kDa HA (HACE_20_) showed favorable phototherapeutic performance and were selected for further in vivo evaluation. In this system, HA contributed to tumor localization and improved the dispersibility of Ce6, while Ce6 served as both the photosensitizer and an optical absorber for fluorescence and PAI. Following intravenous injection into A549 lung tumor-bearing mice, the tumor PA signal progressively increased and was substantially higher than that observed with free Ce6 (Figure 5a), reaching its maximum at approximately 24 h post-injection (Figure 5b). This imaging result was used to identify an appropriate time point for 660 nm laser irradiation. Compared with free Ce6, HACE_20_ nanoparticles exhibited markedly stronger tumor growth inhibition (Figure 5c). After 15 days, the tumor volume in the HACE_20_-treated group was 98 mm^3^, whereas that in the free Ce6-treated group was 730 mm^3^ (Figure 5d). The treatment depth of this system is inherently limited by the absorption properties of Ce6, whose major absorption band around 660 nm results in shallower tissue penetration than that achievable with NIR excitation. Nevertheless, these results illustrate that direct HA–photosensitizer conjugation can improve tumor retention.

**Figure 6:**
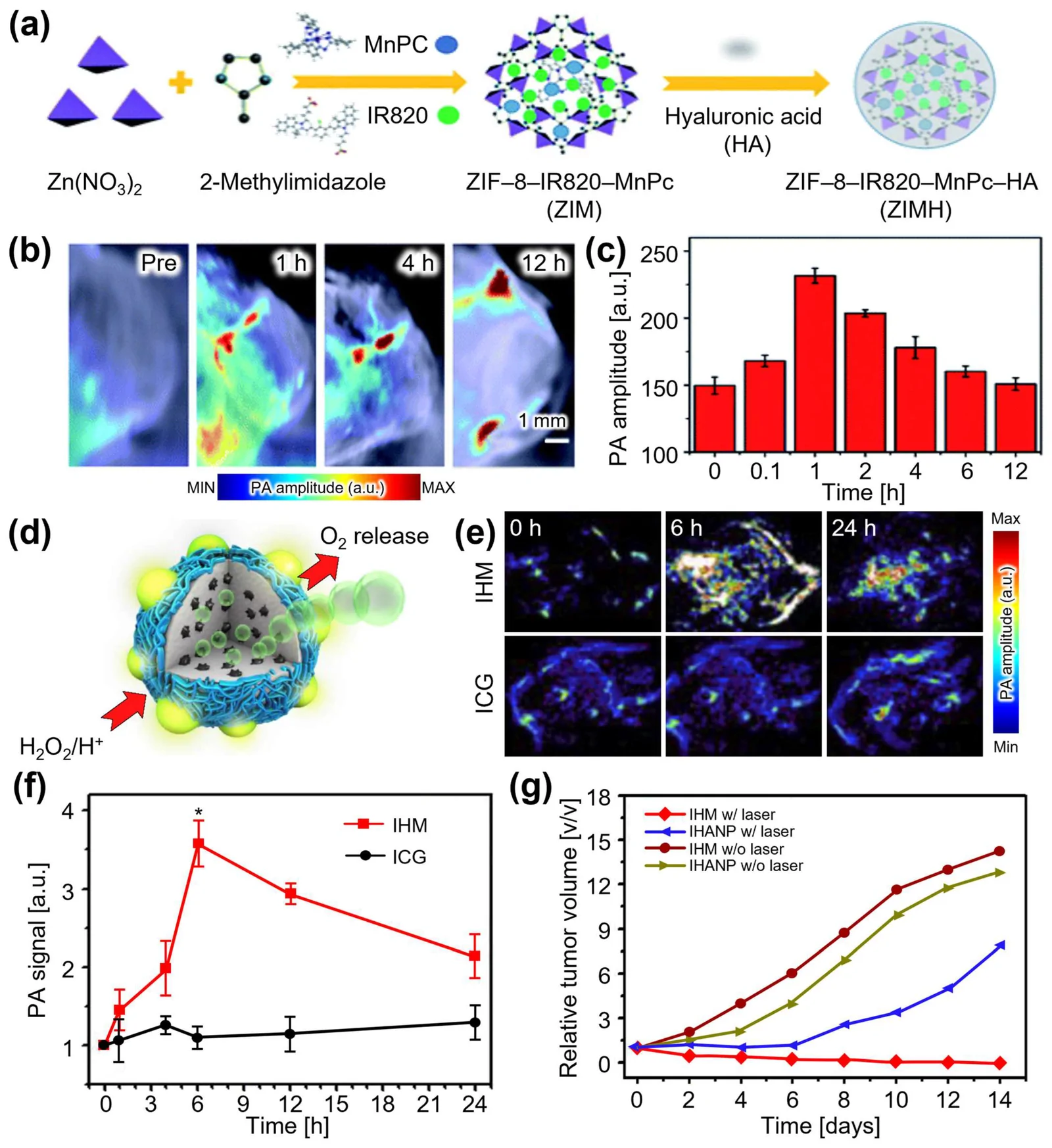
HA-based multifunctional nanoplatforms for PA-monitored PDT. (**a**) Schematic illustration of the preparation of ZIMH nanoparticles. (**b**) Time-dependent in vivo PA images before and after intratumoral injection of ZIMH nanoparticles. (**c**) Quantitative PA amplitude in the tumor region at different post-injection time points. (**d**) Schematic illustration of an oxygen-generating HA nanocomplex containing ICG and MnO_2_, in which MnO_2_ reacts with endogenous H_2_O_2_ to generate O_2_. (**e**) Time-dependent in vivo PA images after intravenous injection of IHM or ICG. (**f**) Quantitative PA signal changes in the tumor region following injection of IHM or ICG. The asterisk indicates the time point at which the peak PA signal was observed. (**g**) Relative tumor volume changes following different treatments, demonstrating enhanced PDT efficacy of the oxygen-generating IHM nanocomplex. HA, hyaluronic acid; ZIMH, HA-coated ZIF-8 nanoparticles co-loaded with IR820 and MnPC; ZIF-8, zeolitic imidazolate framework-8; MnPC, manganese phthalocyanine; Zn(NO_3_)_2_, zinc nitrate; IHM, IHANP/MnO_2_ nanocomplex; IHANP, ICG-modified hyaluronic acid nanoparticle; ICG, indocyanine green; MnO_2_, manganese dioxide; H_2_O_2_, hydrogen peroxide; H^+^, hydrogen ion; O_2_, oxygen; PA, photoacoustic; PDT, photodynamic therapy. The images are reproduced with permission from Refs. [96,97].

Building on this relatively simple conjugate design, more complex HA-coated hybrid nanoplatforms have been developed to assign imaging and therapeutic functions to different components. Qu et al. developed HA-coated zeolitic imidazolate framework-8 (ZIF-8) nanoparticles co-loaded with IR820 and manganese phthalocyanine (MnPC) [96]. In this platform, the porous ZIF-8 framework served as a carrier for encapsulating IR820 and MnPC (Figure 6a). IR820 provided strong NIR absorption for PAI, and MnPC functioned as the photosensitizer for ROS generation under laser irradiation. HA coating was introduced to improve aqueous dispersibility and biological compatibility of the hybrid particles. After intratumoral administration into 4T1 breast tumors, PAI showed a rapid increase in local PA signal (Figure 6b), which peaked at approximately 1 h and gradually decreased thereafter (Figure 6c). The temporal PA profile therefore provided information on the local retention of the nanoplatform and helped define the treatment window. Under 808 nm irradiation, the formulation also generated ROS and produced a photodynamic therapeutic effect. Compared with the direct HA–photosensitizer conjugate, this platform demonstrates how HA can serve as an external functional layer while distinct internal components independently provide PA contrast and photodynamic activity.

Beyond improving photosensitizer delivery and imaging contrast, HA-based nanoplatforms have also been engineered to modify the tumor microenvironment. Tumor hypoxia is particularly important in PDT because insufficient molecular oxygen directly limits ROS generation. To address this limitation, Gao et al. developed an oxygen-generating HA nanocomplex containing indocyanine green (ICG) and manganese dioxide (MnO_2_) [97]. ICG was incorporated as both a photosensitizer and PA contrast agent, whereas MnO_2_ was included to react with endogenous hydrogen peroxide (H_2_O_2_) and generate oxygen within the tumor microenvironment (Figure 6d). HA provided the carrier framework and contributed to tumor localization, while the MnO_2_ component was designed to alleviate local hypoxia and support oxygen-dependent PDT. Following intravenous injection into SCC7 tumor-bearing mice, PAI showed time-dependent tumor accumulation (Figure 6e). The tumor PA signal progressively increased and reached a maximum at approximately 6 h post-injection (Figure 6f). This time point was subsequently selected for laser irradiation. Compared with ICG-containing formulations without MnO_2_, the oxygen-generating nanocomplex produced stronger tumor suppression and sustained therapeutic effects (Figure 6g). The tumors in the treated group were almost completely cured by day 14, with no recurrence observed for 30 days. While this study extends HA-based PDT beyond passive agent delivery by incorporating a microenvironment-modulating component, the tumor selectivity of this system relied mainly on the tumor accumulation of the nanocomplex and localized laser irradiation because ICG remains photoactive wherever it accumulates.

More recently, HA-based PDT platforms have incorporated tumor-responsive activation to improve tumor selectivity and reduce off-target phototoxicity. Fu et al. developed an HA-derived nanoplatform containing a glutathione-responsive phthalocyanine photosensitizer [98]. In the assembled nanoparticle, the photosensitizer was maintained in an aggregated state that favored PA signal generation while suppressing photodynamic activity before reaching the tumor. HA-mediated tumor localization was combined with a sequential activation mechanism in which hyaluronidase-induced carrier degradation and intracellular glutathione promoted nanostructure disassembly and restored the photosensitizing activity of the phthalocyanine. The optimized nanoparticle showed favorable colloidal stability, strong PA contrast, and tumor-selective activation. After intravenous injection into 4T1 tumor-bearing mice, the PA signal became detectable within several hours and reached its maximum at approximately 24 h, providing information on tumor accumulation before irradiation. Subsequent 680 nm laser treatment resulted in greater tumor growth inhibition than non-activated control formulations. PAI in this study primarily reflected nanoplatform accumulation, while photosensitizer activation was confirmed by fluorescence imaging. Despite this limitation, the platform demonstrates a promising strategy that combines PA-visible tumor localization with microenvironment-responsive restoration of PDT, thereby improving therapeutic selectivity.

## 5. Photoacoustic Monitoring of Hyaluronic Acid-Based Nanoplatforms for Combined Photothermal and Photodynamic Therapy

Combined PTT and PDT integrates photothermal conversion and ROS-mediated photodynamic effects within a single therapeutic platform. Because both therapeutic mechanisms depend on the accumulation of optically active agents at the target site, PAI can be used to monitor delivery before treatment. In HA-based combined phototherapy, this capability is particularly useful because a single nanoplatform may incorporate components that simultaneously contribute to PA contrast, photothermal conversion, and photosensitization (Table 3).

**Figure 7:**
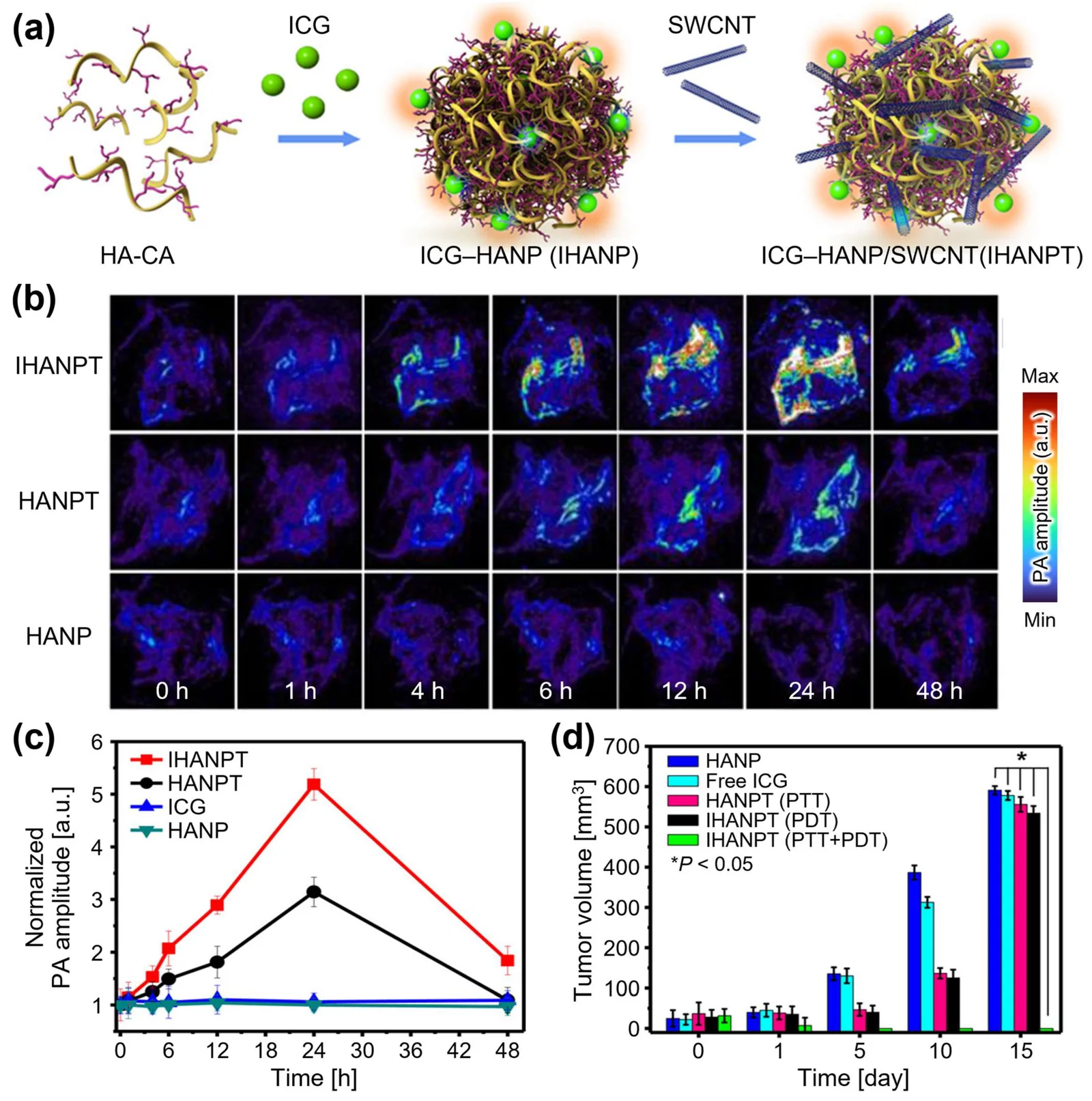
HA-based nanocomplex for PA-monitored combined PTT/PDT. (**a**) Schematic illustration of the preparation of the IHANPT nanocomplex by conjugating ICG to HA nanoparticles followed by incorporation of SWCNTs. (**b**) Time-dependent in vivo PA images after intravenous injection of IHANPT and control formulations. (**c**) Normalized PA amplitude in the tumor region at different time points, showing a maximum signal at approximately 24 h. (**d**) Tumor volume changes following different treatments, demonstrating enhanced therapeutic efficacy of combined PTT/PDT compared with either treatment alone. HA, hyaluronic acid; PA, photoacoustic; PTT, photothermal therapy; PDT, photodynamic therapy; ICG, indocyanine green; SWCNT, single-walled carbon nanotube; HANPT, ICG-conjugated HANP/SWCNT nanocomplex; HANP, hyaluronic acid nanoparticle. The images are reproduced with permission from Ref. [99].

Unlike single-mode phototherapy using either PTT or PDT alone, dual-modal phototherapy that combines both therapeutic approaches generally provides superior therapeutic efficacy through the synergistic effects of PTT and PDT [103,104]. An early strategy for combined PTT/PDT was to integrate a photosensitizer and a strong NIR absorber within a single HA-based carrier. Wang et al. developed an HA-based nanocomplex containing ICG and single-walled carbon nanotubes (SWCNTs) for PA-monitored combined PTT/PDT [99]. The platform was prepared by conjugating ICG to HA nanoparticles and subsequently incorporating SWCNTs into the nanostructure (Figure 7a). In this system, ICG functioned primarily as a photosensitizer while also contributing to NIR absorption, whereas SWCNTs provided strong PA contrast and photothermal conversion. After intravenous injection into SCC7 tumor-bearing mice, time-dependent PAI showed progressive tumor accumulation of the nanocomplex (Figure 7b), with the PA signal reaching its maximum at approximately 24 h post-injection; this time point was selected for 808 nm laser irradiation (Figure 7c). Therapeutic evaluation showed that the nanocomplex- mediated combined PTT/PDT group showed greater tumor suppression than groups receiving either PTT or PDT alone (Figure 7d). These results demonstrate that HA-based nanoplatforms can integrate PAI-based tumor localization with dual phototherapeutic functions under a single NIR excitation. Notably, the laser power density was limited to 0.8 W/cm^2^ to avoid skin damage, and under this condition, PTT alone was insufficient to prevent tumor recurrence. The result highlights a practical limitation of NIR-I phototherapy, in which the amount of optical energy that can be safely delivered to the target is constrained by superficial tissue heating and limited penetration. The addition of PDT therefore provided a complementary therapeutic mechanism that improved treatment efficacy without requiring a further increase in laser power.

**Figure 8:**
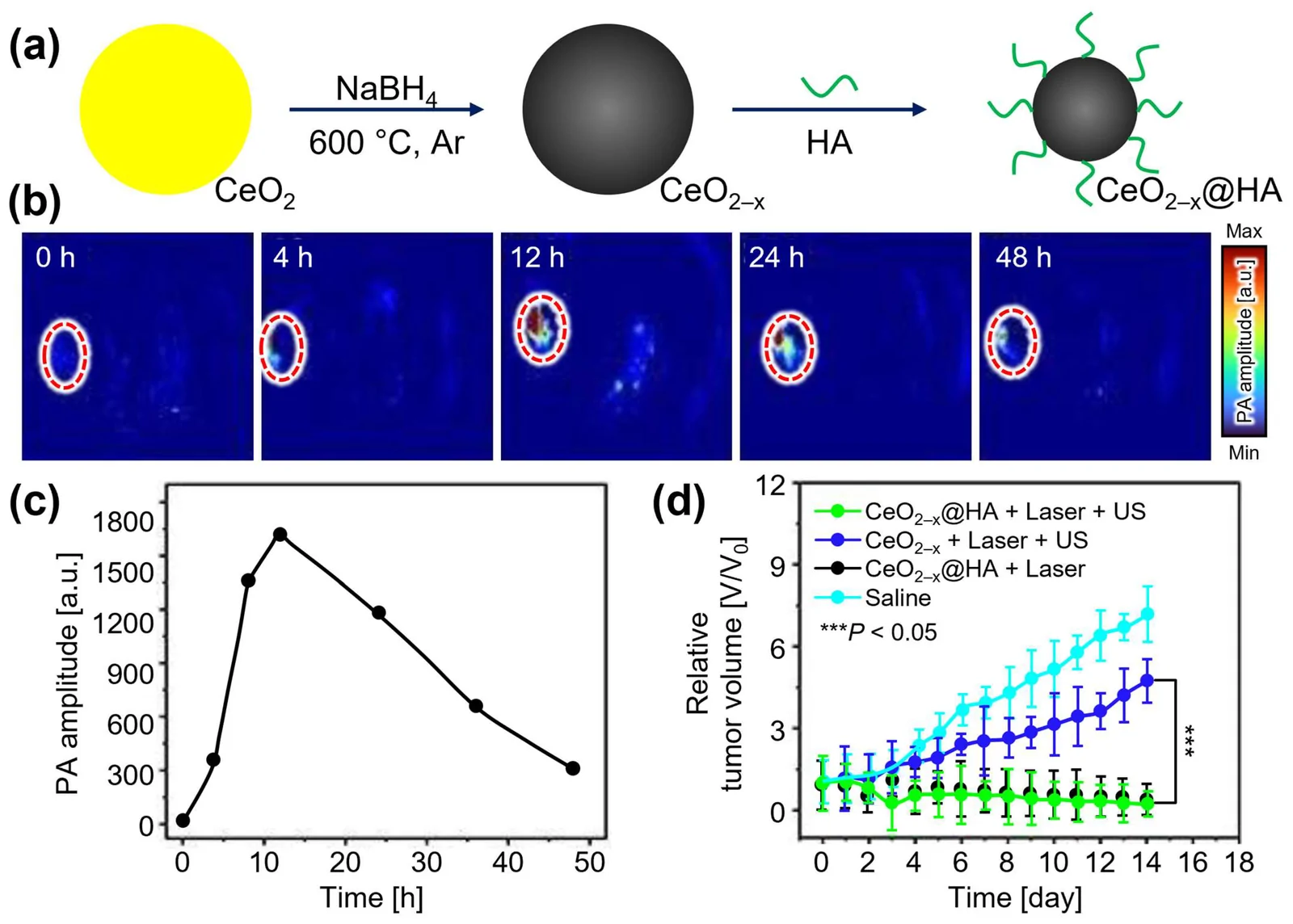
HA-modified CeO_2−x_ nanoparticles for PA-monitored NIR-II PTT/PDT with sonodynamic enhancement. (**a**) Schematic illustration of the preparation of CeO_2−x_@HA nanoparticles by generating oxygen vacancies in CeO_2_ followed by HA surface modification. (**b**) Time-dependent in vivo PA images after intravenous injection of CeO_2−x_@HA. The tumor regions are indicated by red dashed circles. (**c**) Corresponding PA amplitude in the tumor region at different time points, showing a maximum signal at approximately 12 h. (**d**) Relative tumor volume changes following different treatments, demonstrating enhanced tumor suppression by combined NIR-II phototherapy and ultrasound stimulation. HA, hyaluronic acid; PA, photoacoustic; US, ultrasound; CeO_2−x_@HA, HA-modified CeO_2−x_; CeO_2−x_, oxygen-deficient cerium oxide; NaBH_4_, sodium borohydride. The images are reproduced with permission from Ref. [100].

More recently, combined phototherapy has been extended to nanoplatforms responsive to longer-wavelength NIR-II (1000–1700 nm) excitation. Compared with the relatively limited tissue penetration of NIR-I (650–1000 nm) light [105,106], NIR-II light undergoes reduced scattering in biological tissues and permits a higher maximum permissible exposure [107,108], allowing greater optical energy delivery at depth and potentially improving both deep-tissue PA signal generation and phototherapeutic efficacy. Li et al. developed HA-modified oxygen-deficient cerium oxide (CeO_2−x_) nanoparticles for PA-monitored NIR-II phototherapy with additional sonodynamic enhancement [100]. In this system, oxygen vacancies in CeO_2−x_ broadened the optical absorption range into the NIR-II region, enabling PAI and photothermal conversion under 1064 nm laser irradiation (Figure 8a). CeO_2−x_ also generated ROS under 1064 nm laser irradiation, supporting a photodynamic effect, while ultrasound stimulation further enhanced ROS production through sonodynamic therapy. HA surface modification improved aqueous dispersibility and contributed to tumor localization (Figure 8b). After intravenous injection, the tumor PA signal gradually increased and reached a maximum at approximately 12 h post-injection (Figure 8c), which was subsequently selected as the treatment time point. In vivo therapy showed the strongest tumor suppression when HA-modified CeO_2−x_ (CeO_2−x_@HA) was combined with both 1064 nm laser and ultrasound irradiation (Figure 8d). This study extends HA-based PA-monitored combined phototherapy beyond conventional NIR-I excitation by integrating NIR-II PTT/PDT with an additional sonodynamic mechanism.

Using a similar design, Ding et al. developed HA-coated oxygen-deficient tungsten oxide nanoparticles, in which oxygen vacancies enabled broad absorption extending into the NIR-II region [101]. Under 1064 nm irradiation, the nanoparticles generated heat with a photothermal conversion efficiency of 43.6% and produced ROS, while their Fenton-like activity and glutathione depletion provided an additional chemodynamic effect. After intravenous injection into 4T1 tumor-bearing mice, the NIR-II PA signal in the tumor reached its maximum at 12 h, and irradiation at this time point suppressed tumor growth without recurrence over 14 days. These studies extend HA-based PA-monitored combined phototherapy beyond conventional NIR-I excitation by integrating NIR-II PTT/PDT with additional sonodynamic or chemodynamic mechanisms.

HA-based combined phototherapy has also been applied to photoimmunotherapy. Zhang et al. prepared supramolecular nanoparticles by assembling ICG with polyethylenimine-functionalized carbon dots and coating them with HA [102]. Under 808 nm irradiation, the nanoparticles generated both heat and ROS and induced immunogenic cell death in vitro. After intravenous injection into 4T1 tumor-bearing mice, both the fluorescence and PA signals in the tumor reached their maximum at 24 h, and irradiation at this time point increased the tumor temperature to 50 °C. When combined with an anti-PD-1 antibody, the treatment suppressed primary tumor growth by 79%, compared with 58% for phototherapy alone, and also inhibited the growth of distant tumors. This study shows that PAI can monitor combined phototherapy, extending from local tumor ablation to systemic antitumor immunity through photoimmunotherapy.

## 6. Discussion

Recent studies on HA-based PAI-monitored phototherapy have demonstrated the integration of PTT, PDT, and combined PTT/PDT within multifunctional nanoplatforms. Because PA signal generation and phototherapy both originate from optical absorption, many phototherapeutic agents can simultaneously serve as therapeutic components and PA contrast sources. This dual functionality enables noninvasive assessment of agent distribution and tumor accumulation before treatment. In several studies reviewed in this paper, time-dependent PA imaging was used to identify the time point of maximum tumor-associated PA signal, which was then selected for therapeutic irradiation. Therefore, PAI provides a practical solution to link nanoplatform delivery with treatment planning in HA-based phototherapy.

The therapeutic performance of HA-based nanoplatforms depends not only on their targeting and delivery properties but also on the functional design of the incorporated phototherapeutic agents. For both PTT and PDT, efficient delivery to the target tissue, strong absorption at the treatment wavelength, and photostability are important. PTT also requires efficient conversion of the absorbed light into heat. In PDT, ROS generation depends on the properties of the photosensitizer, including its triplet-state quantum yield, as well as the irradiation conditions and surrounding environment. Oxygen availability is particularly important for type II PDT, which produces singlet oxygen through energy transfer [109]. During PDT, the generated ROS may also oxidize the photosensitizer itself, leading to photobleaching and reduced photodynamic activity [110]. Recent developments have therefore included tumor microenvironment-responsive activatable systems, oxygen-generating nanoplatforms, multimodal imaging strategies, combined PTT/PDT, and NIR-II-responsive materials [111,112,113]. Additional modalities, such as sonodynamic therapy, may further extend these systems beyond conventional phototherapy.

An additional advantage of HA-based materials is their potential for localized and transdermal delivery. In addition to intravenous administration, HA can be formulated into topical matrices, hydrogels, and dissolving microneedles that deliver phototherapeutic agents across or through the skin barrier [114,115]. Such approaches are particularly attractive for superficial tumors and dermatological lesions because they can increase local agent concentration while reducing systemic exposure and avoiding repeated injections. The combination of localized HA-mediated delivery with noninvasive PA monitoring may therefore provide a useful strategy for assessing agent distribution and treatment timing directly at superficial lesions.

Several challenges remain for future clinical translation. Most studies have been performed in small-animal tumor models, and further evaluation of long-term biodistribution, biodegradation, clearance, systemic toxicity, and reproducibility is required. HA-mediated targeting should also be interpreted carefully because CD44 expression is heterogeneous across tumor types, disease stages, and individual patients. Therefore, the targeting efficiency of HA-based platforms should be validated for each disease model [116,117,118,119].

From the PAI perspective, most HA-based phototherapy studies reviewed here have used PAI primarily to monitor agent accumulation and to select the timing of irradiation. However, PA amplitude is also affected by optical fluence, tissue depth, acoustic attenuation, detector characteristics, and processing parameters [120], which limits its direct interpretation as a quantitative measure of nanoplatform concentration or therapeutic response. Therefore, standardized PAI protocols and quantitative analysis methods are required to improve the reliability and cross-study comparability of PA-monitored phototherapy.

Future studies should focus on improving quantitative PAI, developing NIR-II-responsive and tumor microenvironment-responsive activatable agents, integrating three-dimensional and multispectral PAI, and validating these systems in tumor models. In parallel, localized and transdermal HA-based delivery strategies may warrant further investigation, particularly for superficial lesions where they may reduce the need for systemic administration.

Multispectral PAI may also provide functional information beyond agent localization by estimating hemoglobin oxygen saturation. This capability is particularly relevant to PDT because therapeutic efficacy depends strongly on molecular oxygen availability, and changes in oxygenation may reflect tumor hypoxia or oxygen consumption during treatment [121]. However, functional oxygenation monitoring has not yet been widely incorporated into the HA-based PDT platforms reviewed here. Future studies that combine agent-specific PA contrast with multispectral oxygenation assessment could provide a more comprehensive evaluation of both nanoplatform delivery and photodynamic response.

Future work should therefore focus on improving quantitative PA imaging, developing NIR-II-responsive and activatable agents, integrating three-dimensional and multispectral PAI, and validating these systems in clinically relevant tumor models. Artificial intelligence-based reconstruction and image analysis may further improve sensitivity, quantification, and treatment-response assessment [122,123]. Together, advances in HA-mediated delivery, multifunctional phototherapeutic design, and quantitative PAI may facilitate the development of more precise and clinically translatable photoacoustic theranostic platforms.

## 7. Conclusions

This review summarized recent advances in HA-based nanoplatforms for PA-monitored PTT, PDT, and combined PTT/PDT. In the reviewed studies, HA improved the stability and delivery of phototherapeutic agents and promoted their tumor accumulation through CD44 binding. When the phototherapeutic agents also act as PA contrast agents, PAI can be used to visualize their distribution and accumulation and to select the time point for laser irradiation. Although promising therapeutic results have been reported, most studies remain at the preclinical stage. Further evaluation of safety, targeting efficiency, and the quantitative accuracy of PA monitoring is needed before clinical application. The combination of HA-based nanoplatforms with PAI may provide a useful approach for phototherapy by enabling both agent delivery and non-invasive monitoring of agent distribution and therapeutic response.

## Figures and Tables

**Table 1:** Summary of HA-based nanoplatforms for PA-monitored PTT. HA, hyaluronic acid; PA, photoacoustic; PTT, photothermal therapy; $\lambda_{th}$, therapeutic wavelength for PTT; $\lambda_{ex}$, excitation wavelength for PA imaging; $t_{peak}$, peak time; FL, fluorescence; CT, X-ray computed tomography; CuS, copper sulfide; Au@Pt, gold-platinum; FePt, iron-platinum; PPy, polypyrrole; AuNR, gold nanorod.

Ref.	Therapeutic/Contrast Agents			Therapy			PA Imaging		Application
	Photothermal Agent	PA Contrast	Other Contrast	$\lambda_{th}$ [nm]	Power [W/cm^2^]	Time [min]	$\lambda_{ex}$ [nm]	$t_{peak}$ [h]	
[82]	CuS	CuS	Cy5.5 (FL)	808	1.5	10	-	6	SCC7 tumor
[83]	Au	Au@Pt	-	808	1.5	10	808	0.5	B16F10 melanoma
[84]	IR825	IR825	Cy5.5 (FL) PFOB (CT)	808	1.5	10	860	24	HT-29 colon tumor
[85]	FePt	FePt	-	808	2.0	5	532–1000	5	MDA-MB-231 tumor
[86]	PPy	PPy	-	808	1.0	5	-	-	4T1 breast tumor
[87]	AuNR	AuNR	-	808	1.0	5	680–850	10	4T1 breast tumor

**Table 2:** Summary of HA-based nanoplatforms for PA-monitored PDT. HA, hyaluronic acid; PDT, photodynamic therapy; PA, photoacoustic; $\lambda_{th}$, therapeutic wavelength for PDT; $\lambda_{ex}$, excitation wavelength for PA imaging; $t_{peak}$, time at peak PA signal; FL, fluorescence; Ce6, chlorin e6; MnPC, manganese(II) phthalocyanine; ICG, indocyanine green; PcG12, glutathione-activatable phthalocyanine photosensitizer; GSH, glutathione.

Ref.	Therapeutic/Contrast Agents			Therapy			PA Imaging		Application
	Photo- Sensitizer	PA Contrast	Other Contrast	$\lambda_{th}$ [nm]	Power [W/cm^2^]	Time [min]	$\lambda_{ex}$ [nm]	$t_{peak}$ [h]	
[95]	Ce6	Ce6	Ce6 (FL)	660	0.16	30	680	24	A549 lung tumor
[96]	MnPc	IR820	IR820 (FL)	808	0.2	5	880	1	4T1 breast tumor
[97]	ICG	ICG	ICG (FL)	808	0.8	10	808	6	SCC7 tumor
[98]	PcG12	J-aggregated PcG12	GSH-activated PcG12 (FL)	680	0.1	10	810	24	4T1 breast tumor

**Table 3:** Summary of HA-based nanoplatforms for PA-monitored combined PTT and PDT. HA, hyaluronic acid; PTT, photothermal therapy; PDT, photodynamic therapy; PA, photoacoustic; $\lambda_{th}$, therapeutic wavelength for PTT and PDT; $\lambda_{ex}$, excitation wavelength for PA imaging; $t_{peak}$, time at peak PA signal; SWCNT, single-walled carbon nanotube; ICG, indocyanine green; CeO_2-x_, oxygen-deficient cerium oxide; WO_3-x_, oxygen-deficient tungsten oxide.

Ref.	Therapeutic/Contrast Agents			Therapy			PA Imaging		Application
	Photothermal Agent	Photo- Sensitizer	PA Contrast	$\lambda_{th}$ [nm]	Power [W/cm^2^]	Time [min]	$\lambda_{ex}$ [nm]	$t_{peak}$ [h]	
[99]	SWCNT/ICG	ICG	SWCNT/ICG	808	0.8	10	-	24	SCC7 tumor
[100]	CeO_2−x_	CeO_2−x_	CeO_2−x_	1064	1.0	10	1064	12	4T1 breast tumor
[101]	WO_3−x_	WO_3−x_	WO_3−x_	1064	1.0	10	1064	12	4T1 breast tumor
[102]	ICG	ICG	ICG	808	0.8	10	-	24	4T1 breast tumor

## Data Availability

No new data were created or analyzed in this study. Data sharing is not applicable to this article.
